# Rat superficial masseter operates at long lengths during biting

**DOI:** 10.1038/s41598-025-21953-z

**Published:** 2025-10-30

**Authors:** Nicolai Konow, Brandon Reder, Daniel Bartlett, Devin Jenness, Trushti Patel, Jeffrey R. Moore, Robert J. Brocklehurst

**Affiliations:** 1 Department Biological Sciences, University of Massachusetts Lowell, Lowell, MA 01854 USA; 2 Monte Rosa Therapeutics, Boston, MA 02118 USA; 3 Jefferson Einstein-Moss Rehabilitation Hospital, Elkins, PA 19027 USA

**Keywords:** Force-length operation, Length-tension relationship, Musculoskeletal function., Evolution, Physiology

## Abstract

The operating length ranges of mammalian jaw muscles have been estimated using twitch contractions or force measurements at the bite point, prompting a consensus that jaw muscles operate at short lengths on their force-length (FL) curve. However, since activation intensity truncates muscle optimal length (*L*_O_), we hypothesized that *L*_O_ of rat superficial masseter (SM) would decrease with activation intensity, with high-force biting involving muscle shortening from long lengths on the FL curve. We measured muscle activation, strain, and force *in vivo* during biting on food with varying hardness and mapped the *in vivo* data from each muscle (*N* = 6) onto its FL relationship, measured *in situ*. Submaximal *L*_O_ was approx. 12% shorter than twitch *L*_O_, and SM bite forces averaged 4.1 ± 3.9 N/cm^2^ (mean ± S.D.) and reached 10.6 N/cm^2^, corresponding to muscle activation and food hardness. Length operation ranged from 7% below *L*_O_ (ascending FL plateau), to 27% beyond (descending limb). The finding that jaw muscles operate at long, potentially unstable lengths, particularly during hard food biting significantly expands our understanding of skeletal muscle function, with broad implications for craniofacial evolution, muscle mechanics and control, and healthy as well as pathological function of the jaw musculoskeletal system.

**Supplementary Information:**

The online version contains supplementary material available at 10.1038/s41598-025-21953-z.

Skeletal movements in vertebrates are driven by muscle contractions, the force generation of which is limited by the overlap of actin and myosin filaments in the sarcomere, in accordance with the force-length (FL) relationship^1–4^. An emerging consensus has been that across scales of biological organization, sarcomeres, fibers, and the fascicles (bundles of fibers) they compose tend to operate at optimal lengths (*L*_O_), centered on the FL curve plateau, where skeletal muscle generates peak force (*F*_O_)^5^. However, under scenarios where stable muscle function is priority, fascicle operating length ranges may be truncated to shorter lengths on the ascending FL limb^6–8^. At such short lengths, additional shortening reduces muscle force as excessive filament overlap causes actin-actin interference or structural alterations and disruptions^2,9^. Conversely, muscle may operate at long lengths, on the descending FL limb^8^. Here, muscle shortening up the descending FL limb, towards the FL plateau increases force, but also involves risks of sarcomere disruption due to the reduced myofilament overlap^10^. Considering the risks of sarcomere disruption, muscles have been thought to only operate at long lengths if they are unlikely to undergo abrupt lengthening. The prevailing consensus on muscle operating length ranges comes from studies of limb and cardiac muscle. Therefore, it remains unclear if the consensus about muscle operating lengths transfers directly to jaw muscle operation or not. Determining whether jaw muscles operate at short lengths, or at long and potentially unstable lengths would significantly expand our understanding of skeletal muscle function. This would also have broad implications for craniofacial evolution, muscle mechanics, and control, and for both healthy and pathological function of the craniofacial musculoskeletal system.

Jaw muscles studies have suggested that force generation during biting at large gapes involve whole muscle and fascicle operating lengths that are short and centered on the ascending FL limb^11–16^. This mode of operation is consistent with the idea that jaw muscles have relatively large moment arms, which result in large muscle length-changes^17^ that inherently could render sarcomeres mechanically unstable. However, jaw muscles rarely experience abrupt lengthening (i.e., eccentric contractions, except in cases of live prey struggling). This musculature would thus be expected to operate at FL plateau-lengths, because FL effects associated with muscle shortening as gape approaches occlusion would reduce force production dramatically^9^. If the SM operates at short ascending limb lengths, force loss during gape closing would be particularly challenging for incisor biting at large gape, as used by many mammals^18^ including rodents. However, approaches in earlier jaw muscle studies, which include force measurements from the entire musculature at the bite point^13^, estimations of muscle length from skeletal displacement, and elicitation of twitch contractions to measure FL from individual muscles^11,14,16^, are unlikely to accurately reflect the patterns of individual muscle activation, contraction, and force generation used during feeding.

Measurements of muscle activation, fascicle strains, and time-varying force production are critical for reliable interpretations of muscle function *in vivo*, for several reasons: (i) The length trajectory of muscle fascicles often differs considerably from the entire muscle-tendon complex, due to the elastic action of compliant tendinous elements^19,20^, and architectural gearing effects caused by fascicles rotating around their tendinous attachments^21–23^. (ii) In most skeletal systems, including jaws, force measured at the contact point can be generated by several muscles whose individual contributions cannot readily be disentangled from time-varying bite-point force^24^. (iii) Muscles are typically activated in a set sequence and at intensities corresponding to the mechanical task. Limb muscle studies have shown that muscle activation intensity impacts the operating length-range of its fascicles^25^. Tetanic activation results in a left-shift of the FL plateau by 10–15% relative to the *L*_O_ of a FL curve generated with twitch contractions^26,27^. However, a general lack of *in vivo* measurements of muscle activation, fascicle strains, and time-varying force generation from individual muscles of the jaw means that we do not know if they undergo the same activation-dependent operating length-shift. It also remains unknown what the FL effect on jaw muscle operation might be. We hypothesize that conclusions drawn in earlier jaw studies, suggesting that muscles of the jaw operate at relatively short lengths^11,14,16^, were influenced by the twitch activation protocol used. We predict that operation at short twitch lengths actually correspond to operation on the FL plateau or longer ascending limb lengths under greater (submaximal tetanic) activation conditions, as necessary for generating sufficient bite force.

To determine if earlier study protocols led to an imprecise consensus about the length-operation range of jaw muscles, we elicited incisor biting in rats feeding on an assortment of natural food items. We used food types that rats willingly accept with material properties, including systematic hardness differences that have been thoroughly characterized, to facilitate comparisons of our data with existing literature^28,29^. We used direct instrumentation of the rat superficial masseter (SM) to collect direct measurements of time-varying force production from a major jaw closer muscle during feeding, along with electromyographical measurements of muscle activation and recruitment (EMG), and measurements of fascicle length-change dynamics (sonomicrometry). We then tested the same muscle preparations with an *in situ* ergometry experiment that measures twitch and submaximal tetanic FL. Finally, we mapped *in vivo* fascicle length-operating ranges and forces to the *in situ* FL curves^8,20,30^. For limb muscles, this approach has proven powerful by determining *in vivo* constraints on muscle force and fascicle length, which we have previously only indirectly achieved for jaw muscles^9^. This experimental design allowed us to test the hypothesis that large fascicle strains in SM, associated with incisor biting at large gape, lead to peak force generation at fascicle operating lengths that correspond to the descending limb on the submaximal tetanic FL curve, in contrast to the findings of earlier studies that used twitch contractions.

## Results

**Fig. 1:**
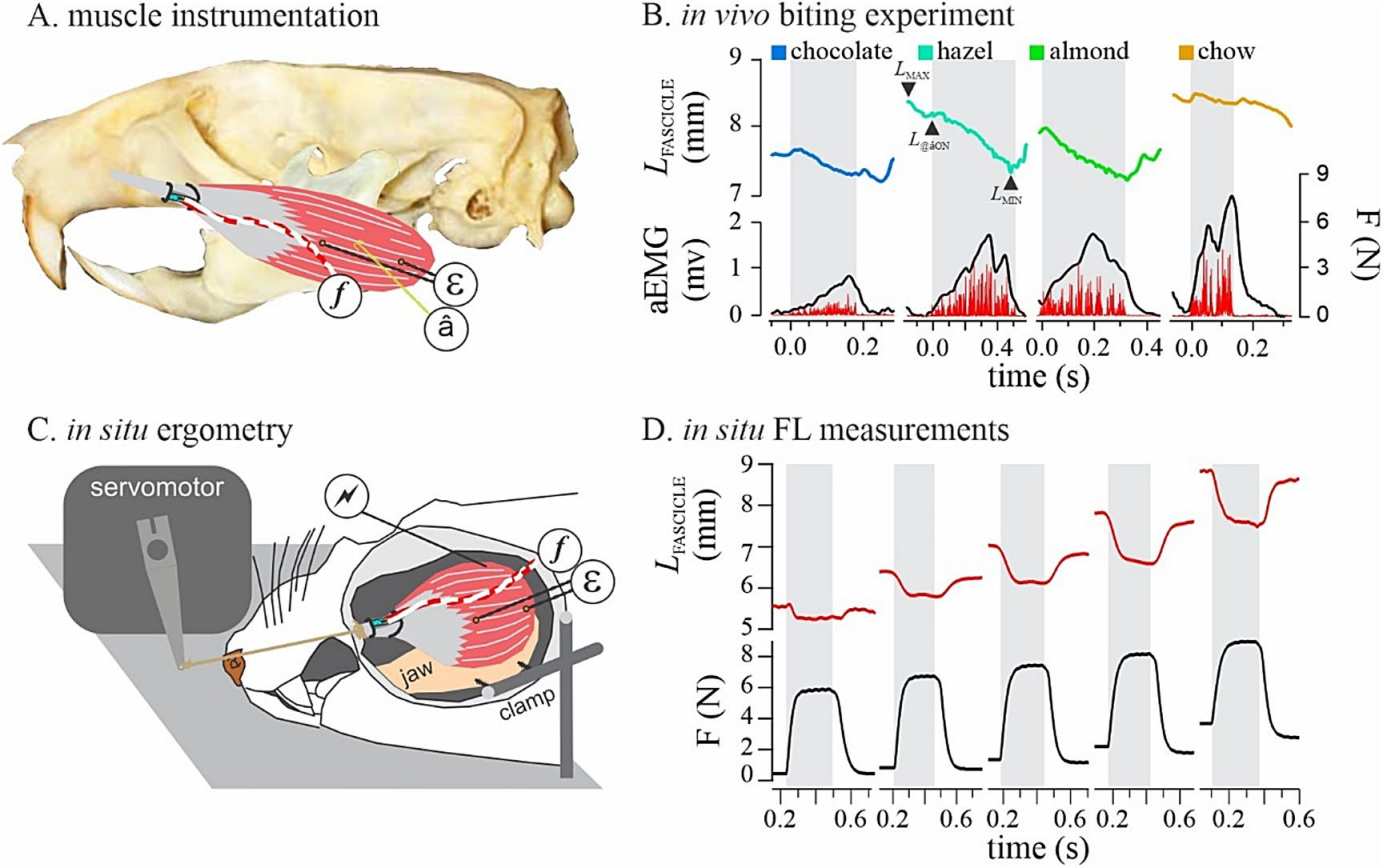
Instrumentation and experimental protocols. (**A**) The unipennate rat superficial masseter (grey is tendon, red is fascicles), instrumented with EMG to measure muscle activation (â), sonomicrometry to measure length-change of muscle fascicles (ε), and a custom miniature strain-gauge buckle to measure muscle force ( *f* ). (**B**) Representative *in vivo* measurements of muscle activation, length, and force for incisor biting on food with varying hardness (color). Grey bars delineate biting power strokes. Note how starting length (*L*_MAX_) can exceed activation length (*L* @_âON_) due to earlier activation of other adductor muscles. (**C**) The muscle preparations tested *in vivo* were also tested *in situ* using an ergometry procedure to measure muscle FL with needle stimulation (, 250ms for submax tetanic, and 2ms for twitch). (**D**) Representative contractions used to construct the muscle’s FL relationship. Grey bars indicate stimulation intervals.

Across the six rats tested *in vivo* in biting experiments (Fig. 1A, B), we were not always able to collect reliable bites for all food types, and a total of 247 bite recordings were used for analyses (see Supplemental Table 3).

**Fig. 2:**
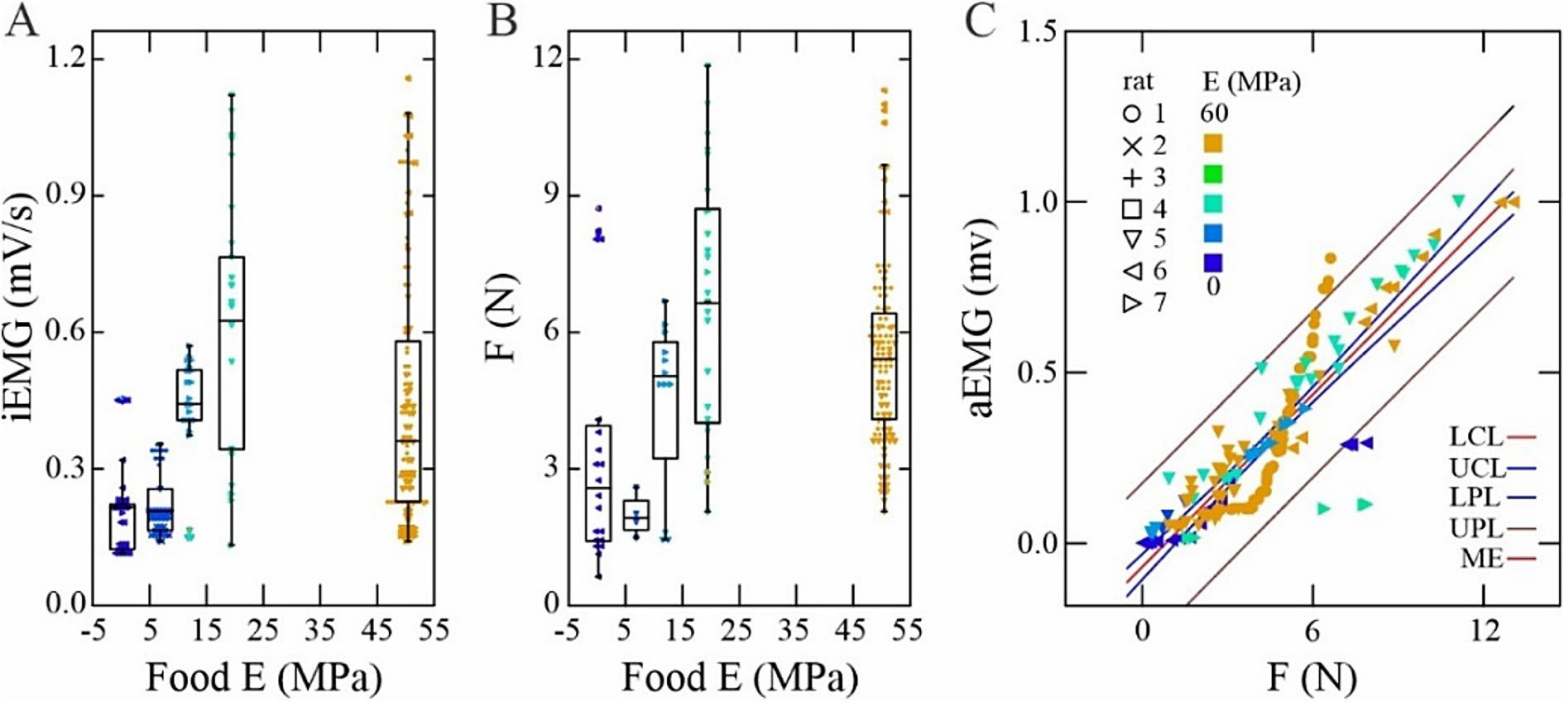
Influence of food hardness on muscle recruitment and force production. During rat incisor biting, greater food hardness increases superficial masseter (**A**) electromyographic recruitment, and (**B**) force production. (**C**) A strong relationship between EMG intensity and force production exists across rats (symbols) and food hardnesses (colors) (For Linear Least-Squares regression results, see methods. LCL, UCL, Lower and Upper Confidence Limits; LPL, UPL, Lower and Upper Probability Limits; ME, Model Estimate).

We found a strong relationship between recruitment of rat SM and its force production across incisor bites on foods with varying hardness. Food hardness correlated strongly with muscle recruitment intensity (GLM: F_2,236_ = 10.8; *p* < 0.0001; η^2^ = 0.32), measured as the integrated area under the rectified EMG signal (Fig. 2A). Harder food was associated with greater muscle force production (Fig. 2B), again with a statistically significant relationship (GLM: F_2,161_ = 4.8; *p* < 0.01; η^2^ = 0.07). Whereas EMG activity and force production generally increased with food hardness, this relationship plateaued at levels of hardness above 20 MPa. Rat chow, nominally the hardest food tested (50.44 MPa;^29^), elicited lower-than-expected EMG and force production compared to predictions based on muscle force production on softer foods.

Stress developed *in vivo* by the rat SM increased considerably with food hardness and averaged 4.1 ± 3.9 N/cm^2^ (mean ± S.D.). Greater stresses were consistently recorded for the two hardest food items (almond and chow) and peaked for almond at 10.6 N/cm^2^, with median stress for chow bites (7.2 ± 3.9 N/cm^2^) being marginally lower than for almond bites (8.6 ± 5.3 N/cm^2^) (F_2,125_ = 4.966; *p* < 0.05). Based on an average muscle PCSA of 0.51 ± 0.12 cm^2^ (Supplemental Table 1), and specific tension data for vertebrate skeletal muscle^31,32^, the rat SM operated at submaximal recruitment during incisor biting. A Linear Least-Squares regression of EMG recruitment against force production indicated a strong EMG-force relationship (F_1,161_ = 516.9; *p* < 0.0001), despite identifiable variation across subjects and food hardness (Fig. 2C).

**Fig. 3:**
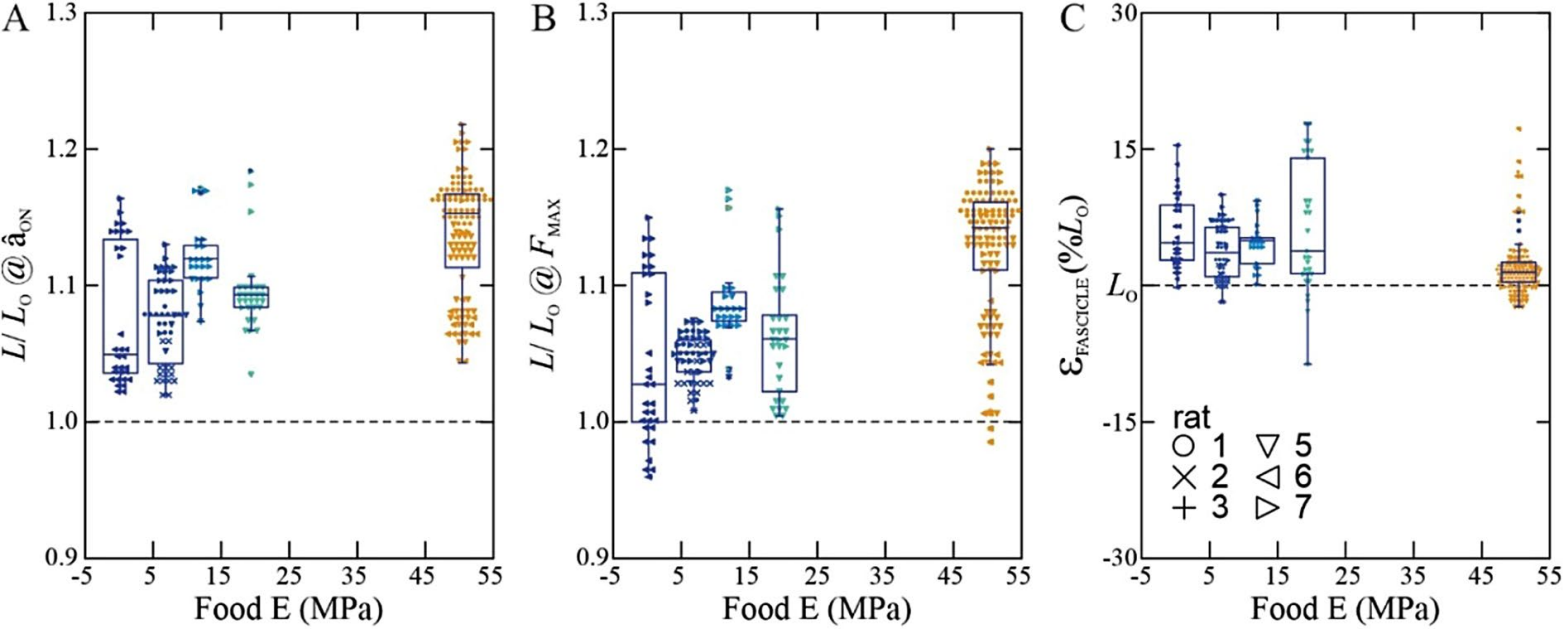
Fascicle in rat SM operate at long relative lengths (**A**, **B**) and small strains (**C**) during incisor-biting. Fascicle relative lengths (*L*/*L*_O_) at activation onset (**A**) and coincident with peak force (**B**) increase with food hardness (color scheme as in Fig. 2). Fascicle strains (Ԑ) averaging 5–7% across food types are consistent with the pennate architecture of SM, and with operation beyond the FL plateau^5^.

The operating length-ranges of fascicles in the rat SM were strongly correlated with food hardness. Peak relative lengths (*L*_MAX_) typically occurred at bite onset (Fig. 1B) and preceded as well as exceeded relative fascicle length at activation (*L* @â_ON_), likely due to earlier activation and action of different adductor muscles in the rat feeding system^33^. Increased food hardness elicited longer relative fascicle lengths at muscle activation onset (*L*/*L*_O_ @â_ON_) (GLM: F_2,237_ = 52.1; *p* < 0.0001; η^2^ = 0.98) (Fig. 3A) and at occlusion (*L*/*L*_O_ @F_MAX_) (F_2,237_ = 74.4; *p* < 0.0001; η^2^ = 0.68) (Fig. 3B). The strain range for contracting fascicles varied considerably (Fig. 3C) and averaged 10.5 ± 3.2%, with a statistically significant reduction in strain with increasing food hardness (F_2,237_ = 24.8; *p* < 0.0001; η^2^ = 0.69).

**Fig. 4:**
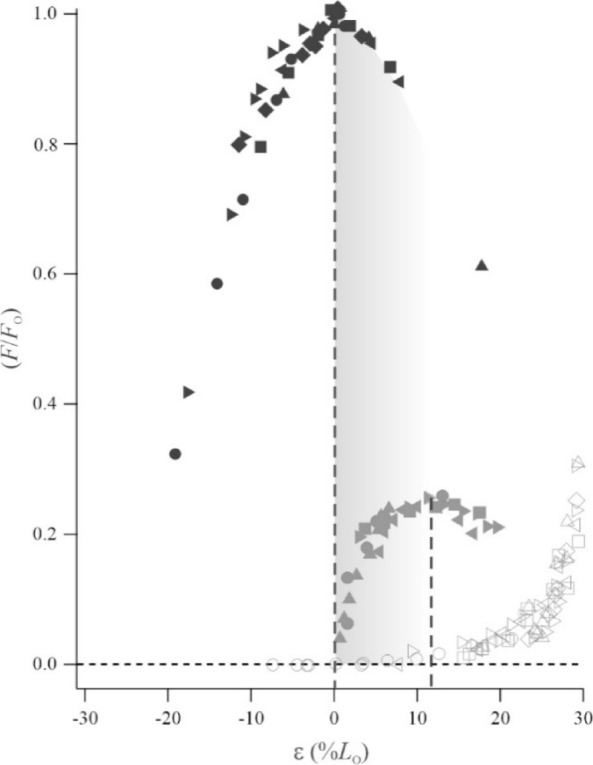
In rat SM, the plateau of the relationship between relative force (*F*/*F*_O_) and strain (%*L*_O_) is left-shifted with increased muscle activation. Submaximal tetanic *L*_O_ (dashed vertical line to black filled symbols) is left-shifted by 11.3 ± 2.9% from twitch *L*_O_ (dashed vertical line to grey filled symbols), consistent with measurements from limb muscles^26,27,34,36^. Open grey symbols show the passive FL relationship. The shaded area highlights how the operating length range of twitch-activated muscle on the ascending twitch FL limb is equivalent to the operating length range of submaximally-activated muscle on the descending submaximal FL limb.

Our *in situ* preparations of rat superficial masseters (Fig. 1C, D) achieved peak tension development under twitch activation at optimal length (*L*_O_) reaching 12.3 ± 4.3% relative to tetanic activation (*F*_O_). Tetanic tension at *L*_O_ averaged 10.1 ± 3.3 N/cm^2^, suggesting that our needle stimulation protocol elicited submaximal and not maximal tetanic contractions. Comparisons of *L*_O_ for twitch and submaximal tetanic FL curves showed that the submaximal tetanic plateau was left-shifted by 11.3 ± 2.9% with respect to the twitch plateau (Fig. 4).

**Fig. 5:**
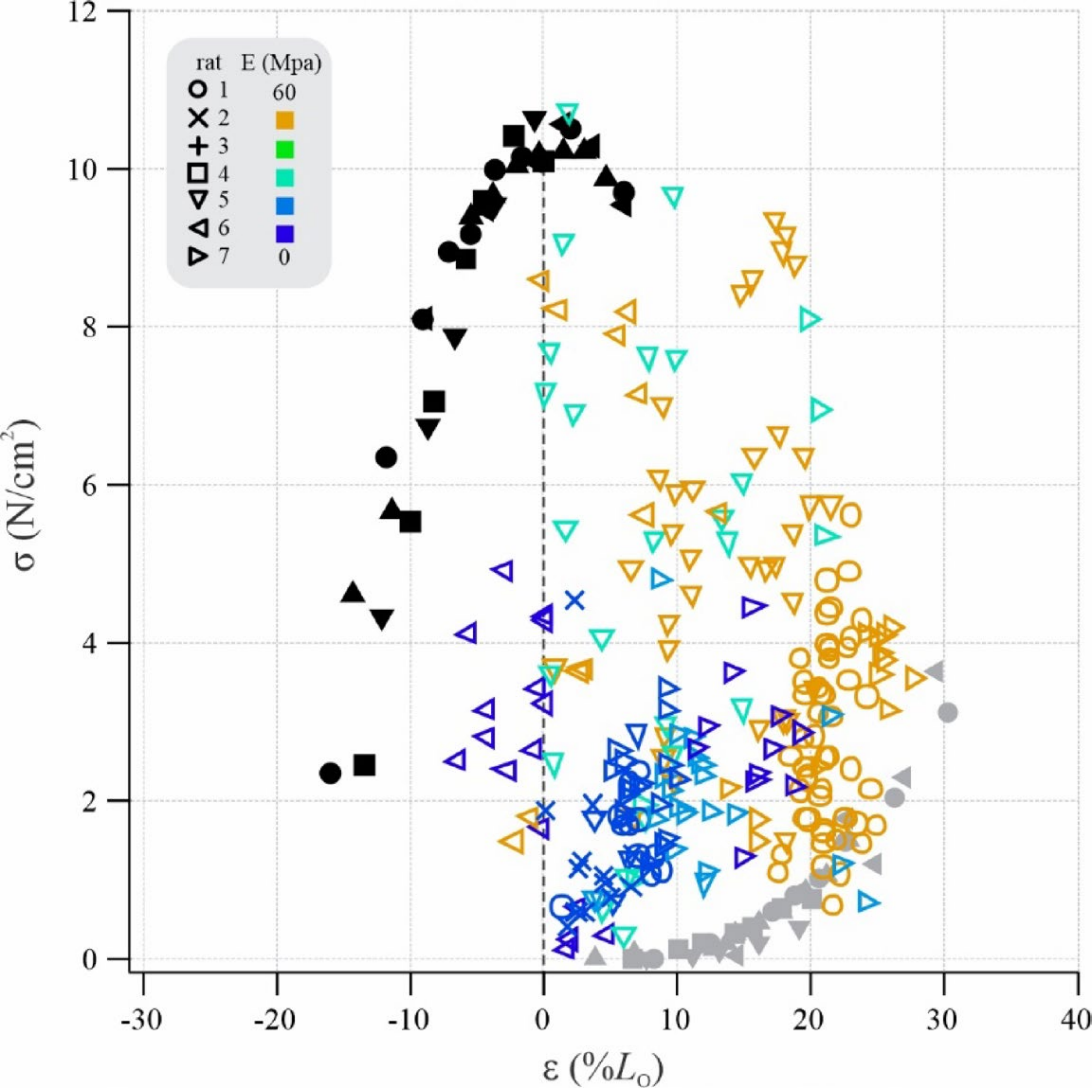
Plateau and descending FL limb operation of rat SM during incisor biting. Superimposition of *in vivo* stress-strain operation of rat SM (colored symbols are data from individual bites) onto the passive (grey) and active (black) stress-strain relationships obtained *in situ* under submaximal tetanic stimulation. Bite data are peak stress (σ) plotted against fascicle strain (ε) at peak force production near occlusion, typically being the shortest fascicle length reached during biting (Fig. 3B). Note how bites on the hardest food type (chow, E = 50.4 MPa, orange) occur at long strains on the descending FL limb, whereas bites on the softest food type tested (raisin, E = 0.2 MPa, purple) involve low stress and occur at FL plateau lengths.

Superimposition of *in situ* and *in vivo* data from rat SM (Fig. 5) revealed incisor biting to involve fascicle operating strain-ranges spanning from in excess of 25% above *L*_O_, on the descending FL limb (i.e., high ε) across the FL plateau to approximately 7% down the slow ascending FL limb (i.e., low ε). Both hard and soft food types elicited bites involving high fascicle peak strains (ε _MAX_). However, fascicle operation at low strains were particularly evident for soft food types and fascicle operation at high strains were particularly common for hard food types, which also elicited the greatest tension generation as the jaw approached occlusion.

## Discussion

Our *in vivo* study of the rat superficial masseter during incisor biting generated the first available ensemble measurements of electromyographical activation, fascicle strain, and force from a jaw muscle during feeding. We mapped the *in vivo* operating stress and strain ranges of the same muscle onto its submaximal force-length (FL) relationship, as measured from the same muscle preparations *in situ*. Combined, these data revealed that as activation increases, the FL plateau of rat superficial masseter (SM) shifts leftward to a similar extent as previously reported for various limb muscles. Taken together, our data show that the rat SM operates *in vivo* across lengths that range from the descending FL limb, across the plateau, and down the ascending limb. This finding runs counter to the well-established paradigm that jaw muscles operate at short, stable lengths^11–16^. Our findings further develop our understanding of how skeletal muscle exploits the FL relationship *in vivo* to maximize performance for specific tasks, in this case biting. These results highlight the role of muscle operating length in maintaining musculoskeletal stability during routine motions. Our finding has broad implications, ranging from reconstruction of craniofacial evolution in vertebrates to the prevention and treatment of temporomandibular joint disorders (TMD) in humans.

Muscle operating length depends on the intensity of muscle activation and recruitment. For the rat SM, we discovered a left-shift of the plateau of the muscle’s FL curve averaging 11.3 ± 2.9% *L*_O_ as we increased stimulation voltage *in situ*, from twitch to submaximal tetanic stimulation. This shift in optimal length (*L*_O_) is consistent with earlier experiments that varied stimulation voltage to elicit twitch, submaximal tetanic, and maximal tetanic contractions in limb muscles, including toad plantaris (> 15% *L*_O_^34^), Guinea Fowl lateral gastrocnemius (20% *L*_O_^35^), rat medial gastrocnemius (12.6–11.2% *L*_O_, across muscle compartments^27^), and mouse soleus and tibialis anterior (both 8.2% *L*_O_^26^). For cat soleus, a similar shift was demonstrated by varying stimulation frequency (25% *L*_O_^36^). Our discovery of a plateau-shift for a jaw muscle that is comparable to measurements from limb muscles with a diversity of fascicle architectures, series elastic element prominences, and mechanical functions suggest that this trait is generalizable across striated skeletal muscle. The mechanistic explanation for the plateau-shift remains incomplete as both strain-dependent changes in calcium sensitivity affecting cross-bridge cycling^25,36–38^ and series elastic compliance^38–40^ have been implicated. Regardless, our findings from rat SM suggest that studies using twitch activation to examine FL behavior in jaw muscles^14,16^ have generated an inaccurate consensus about the lengths across which jaw adductor muscles operate. Our conclusion is strengthened by the consideration that muscles rarely are twitch-activated nor supramaximally activated *in vivo* and typically operate at submaximal levels of activation and recruitment^27^.

The central nervous system regulates muscle activation timing and both the intensity and completeness of muscle recruitment to changes in loading environment and force requirements^41–43^, for instance, as elicited by changes in food hardness. In agreement with this principle, we found a strong positive relationship between the recruitment intensity (integrated area under the EMG) of the rat SM during *in vivo* incisor biting power-strokes with the hardness of the food substrate (LMS regression, *p* < 0001). A similar strong positive relationship was also established between muscle force output and food hardness, with few exceptions (as discussed below). Overall, our findings indicate a generally strong agreement between EMG and force measurements, consistent with recordings from a diversity of limb musculoskeletal systems^44,45^. This finding suggests that EMG holds promise for understanding time-varying force production in other jaw muscles, where substrates for force-buckle attachments are unavailable, such as the pterygoids and temporalis.

As food hardness was increased during rat *in vivo* biting, we found systematic changes in the operating length ranges of the muscle fascicles, as measured directly using sonomicrometry. As muscle recruitment intensity and force production increased with food hardness, muscle fascicles operated across longer length-ranges but also contracted to relatively shorter lengths (Fig. 3B; GLM; both *p* < 0.0001). These findings underscore that muscles can exploit FL effects by tuning operating length to generate forces that match task-based requirements, contend with length-based limitations to force generation, and mitigate risks of contracting at long lengths^8,9^.

To better understand how the rat SM exploits its FL relationship during feeding, we superimposed our *in vivo* and *in situ* measurements. This is a validated and powerful method for rigorously interpreting operating requirements and constraints on skeletal muscles in limbs^20,35,46^. Mapping the *in vivo* operating length ranges of rat SM onto the muscle’s own FL relationships, as measured *in situ* allowed testing of the hypothesis that twitch FL studies inaccurately reflect the muscles’ contractile behavior. In line with earlier twitch studies of jaw muscles^14,16^, we determined that the *in vivo* operating length-ranges of the rat SM during incisor biting corresponded to the ascending limb (i.e. short operating lengths) of the twitch FL curve. However, when the *in vivo* operating length ranges were mapped to the significantly shorter plateau-lengths of the submaximal FL curve, with comparable stresses, we found that the realized operating lengths were typically distributed across the FL plateau. For bites on hard food (almond, chow) the muscle often operated at relatively high stresses on the descending limb (i.e., long lengths). This discovery is important for several reasons. Limb muscles will often, but not always, operate across the FL plateau where muscle force is maximized^8,47^. Our data, therefore, suggest general similarities between limb and jaw muscles in this regard (see also^9^).

We tested natural foods that rats would willingly accept and attempted to size-match food items to avoid food size-driven FL effects in our *in vivo* data. Evidence that our attempts to decouple muscle force from gape and fascicle length were not entirely successful is seen for our hardest food type, chow (E = 50.44 MPa^29^). Chow elicited significantly lower muscle recruitment intensity and force production (Fig. 2) than our second-hardest food type, almond (E = 19.4 MPa). This result may be influenced by uncontrolled food properties such as moisture content, with which hardness likely co-varies^48^ as well as the granular nature of chow that may afford the incisors easier purchase, and also fractures more easily between incisors than between materials testing plates. However, food-driven FL effects were likely not completely avoided as chow pieces invariably were larger than raisins. Consequently, length effects on muscle function, driven by variations in food size^9^ likely also play a role because relative fascicle operating lengths were considerably longer for chow than for other food types (Fig. 3A, B). Long fascicle lengths may have prevented greater force generation on chow (Fig. 2A, B), although the associated depression in force likely was somewhat offset by force-velocity effects^46^. For example, muscle operating strains on chow were generally shorter (Fig. 3C) and slower (Fig. 1B) than for other food types. Conversely, our softest food type, raisin (E = 0.22 MPa) elicited greater-than-expected muscle forces (Fig. 1B) and more variable operating lengths (Fig. 2A, B) than other food types. This result may be caused by the greater compliance (i.e., stress to fracture) of raisins, requiring greater biting work than other food types tested (Supplemental Table 2). Together, the anomalous force-responses by SM on the nominally softest and hardest food type respectively may be the reason that the influence of food hardness on SM force production carried the lowest effect size (η^2^ = 0.07) of our statistical comparisons.

Future work building on ours could use manufactured food items that permit more careful control and parameterization of shape, size, and hardness^9,49^ than natural foods do. Analyses could include single bite interactions on spherical food with controlled variations of size and/or hardness. Such analyses would also benefit from videofluoroscopic imaging^9^ to precisely determine jaw gape and where the incisors make purchase on the food item. They should also incorporate skeletal and muscle moment arm analyses^17^, especially considering that the rodent jaw joint anatomy allows propalinal (caudal-to-rostral) jaw translation during gape closing, which likely affects muscle operating length independent from vertical gape movements.

Foods used in our study are unlikely to have challenged the rat jaw apparatus, which for instance is capable of fracturing pistachio shell (E = 1.2 GPa; Pers. Obs.). Accordingly, forces measured *in vivo* were unlikely representative of the peak voluntary contractile capacity of the rat SM, which may be realized not only during feeding but also during defensive biting. Here, the maximal FL curve would be relevant, and our specific tension calculations suggest that the maximal FL plateau would be left-shifted by 10% from submaximal *L*o, placing peak bite force events on the deep descending FL limb. We were unable to elicit supramaximal contractions *in situ* due to the surgical infeasibility of accessing the nerve of masseter for mounting of a cuff electrode that would facilitate supramaximal stimulation of the rat SM. However, given the well-matched peak tension recorded *in vivo* during biting and elicited in our *in situ* tetanic contractions, the methodological limitations of needle electrode stimulation are unlikely to affect the conclusions of our study. Despite the good agreement between the specific tension values we recorded *in vivo* and the peak tension elicited *in situ*, future studies should explore alternative stimulation approaches^14^ to elicit supramaximal stimulation and generate *in situ* FL data that match supramaximal behaviors.

The discovery that the rat SM operated at long fascicle and sarcomere lengths to generate sufficient force to fracture hard food is an interesting finding from the perspectives of muscle as well as jaw joint stability and safety. Compared to short fascicle and sarcomere length operation, where muscle is stable^6^, large and hard food items drove the muscle to develop high forces at long lengths where inherent sarcomere instability due to weak actomyosin overlap^2^ might lead to muscle damage^10^. The dangers of the descending FL limb have been highlighted for limb muscle^50^ but may potentially be greater for jaw muscles due to their larger moment arms^17^. If food item slippage was to translate the jaw during high force production at large gape, the resulting stretch of the muscle might compromise its ability to counter dislocating torques at the temporomandibular joint (TMJ). Other potential deleterious results from such an event include damage to the dentition^16^. Jaw muscles in mammals tend to alternate between powering and balancing the jaw during feeding^24,51,52^. Accordingly, muscle instability might trigger jaw joint instability and our data suggest that biting on large and hard objects could be an overlooked trigger of dysphagia including temporomandibular joint disorders (TMD). We anticipate future FL operation analyses of mammalian jaw muscles to inform research on healthy temporomandibular joint (TMJ) function as well as triggers of its dysfunction.

Our data on the effects of food types with different material properties echo previous work by paleontologists and anthropologists seeking to reconstruct foraging and feeding in our mammalian and human forebearers^28,29^. However, such studies have previously lacked the analytical power that single muscle force measurements provide^20,51,55^. The force-length effects highlighted by our data are especially relevant for computational modeling of the craniofacial system, where the force-length relationships of individual muscles often are greatly simplified and do not incorporate factors such as activation-dependent shifts of optimal length^53–55^. Computational models are a common tool given the challenging nature of experimental work on primates (including humans) and are the only tool available for studying craniofacial evolution in the fossil record. Several key transitions in vertebrate evolution involve changes to the mandible and jaw joint, where continued functioning and stability of the system would be paramount e.g., mandibular reduction in the lineage leading to modern humans, and the transformation of the post-dentary bones into the mammalian middle-ear ossicles^56,57^. Our experimental data demonstrate a clear need to fully understand jaw biomechanical systems in order to inform modeling approaches aimed at accurately reconstructing key functional transitions across major clades.

## Methods

Sprague-Dawley rats (*N* = 10, 50/50 M/F) were purchased from Charles River Laboratories and same-sex pair-housed under a 12 H light : 12 H dark cycle at the Concord Field Station (Harvard University). All methods were carried out in accordance with relevant guidelines and regulations. Methods are reported in accordance with ARRIVE guidelines. Both husbandry and experimental procedures adhered to protocols approved by the Institutional Animal Care and Use Committees at Harvard University (FAS, 20 − 09_04) and UMass Lowell (23 − 03). Rats were provisioned with water and rodent chow *ad libitum* and at the time of experiments, body masses were 440 ± 113 g (mean ± S.D.) and ages were 13.5 ± 1.8 weeks (mean ± S.D.) (Supplemental Data Table 1). Power analysis based on muscle activation, strain, and force data from our pilot study suggested that *N* = 5 would yield a Delta (mean difference over individual variation) for two-factored GLM designs of 1.8–2.5 and analysis power ranging between 87% and 96% at α = 0.01 (Systat v. 12).

**Table 1:** Summary data from *in situ* experiment on rat superficial masseter.

Rat	Stim (V)	F_O_ (*N*) (submax)	F_O_ (*N*) (twitch)	L_O_ (mm) (submax)	L_O_ (mm) (twitch)	Tension (submax)
1	10	6.5*	-	12.8	-	10.5
2	9	6.7*	-	8.7	-	14.1
3	10	5.7	0.4	6.1	6.7	12.8
4	11	6.6^#^	-	-	-	12.3
5	6	3.5	0.7	8.5	9.6	6.9
6	10	3.7^#^	-	-	-	7.3
7	6	6.2	1.9	5.2	6.2	8.1
8	12	6.2	1.5	3.1	3.4	11.1
9	8	7.3	2.2	5.8	6.1	9.0

*Twitch FL not measured. ^#^FL curves from motor position, not fascicle length.

### Instrumentation surgery

Rats (*N* = 6) were induced in a chamber on isoflurane (4-4.5%) via oxygen (2.0 L/min) and transferred to a low-profile mask for anesthetic maintenance at 1.5–2.5% isoflurane via oxygen (0.8 L/min). Core temperature was monitored via a rectal thermocouple and adjusted using an infrared lamp and a controllable heat pad. Breathing rate and core temperature were continuously monitored and recorded at 10 min intervals throughout each procedure. After application of ophthalmic ointment and injection of 2 ml sterile saline (SQ), incision areas were shaved and sterilized using three repeated alternating scrubs of chlorhexidine and betadine. The subject was draped with semitransparent plastic (Press’n Seal) to allow visual monitoring of breathing during the sterile implantation procedure that followed.

Two skin incisions were made, one along the left lateral-to-ventral chin and one along the mid-dorsum of the neck to route wires subcutaneously through a sterile plastic straw, from a skin port (Microtech, Bronwyn PA) attached at the dorsal incision, to the chin incision, over the SM (Fig. 1A). A pair of sonomicrometry crystals (1 mm, Sonometrics Corporation, London ON) were implanted into the SM muscle along the length of a fascicle. One crystal was implanted at the myotendinous junction, and one at the mandibular origin. The distance between the crystals were 12 ± 3 mm. Crystals were anchored to the epimysium using 6 − 0 silk and oriented in the tissue to generate the strongest possible transducer pulse (monitored during surgery via an oscilloscope). To measure EMG, a bipolar, offset-hooked electrode^58^ (California fine wire, Grover beach, CA) was implanted using a 25G hypodermic needle mid-belly into the muscle, along the same fascicle as the sonomicrometry crystals. The EMG electrode was also sutured to the epimysium using 6 − 0 silk. A custom-made miniature strain buckle (FLK-1–11, Tokyo Sokki Kenkyujo)^59^ was tied to the tendon of SM using 3 − 0 silk. The rat and its instrumentation was grounded using a large-gauge blunt monopolar steel electrode located under the skin port chassis. Both skin incisions were closed using 3 − 0 Vicryl. Flunixin Melgumine (30 mg/kg, SQ) was administered as pain relief and analgesics intraoperatively, and at 12 h intervals throughout recovery. Once awakened from surgery, subjects were fed soft (raisin) food within the hour to ensure recovery to normal behavior, and then fasted during recovery (overnight, 12–16 h) to elicit optimal feeding performance during the *in vivo* feeding experiment.

### *In vivo* feeding experiment

Our *in vivo* experiment was designed to test bite performance across a range of food types with known hardness (Range of Young’s Modulus; 0.22–50.44 MPa;^28,29,60^; Supplemental data Table 2) and near-equivalent size (approx. 1.5 cm diameter). We avoided mechanical alteration of food size due to concerns that this would affect the opportunities for the subject’s incisors to make purchase on the food during biting. During experiments *in vivo*, the subject was placed in a transparent acrylic enclosure (25 × 25 × 25 cm). The data collection cable was connected to the skin port and the recording equipment and freely suspended above the subject’s neck using nylon string. The rat was presented with food items (Supplemental data Table 2) in a pseudorandomized sequence from a sturdily attached, spring-loaded metal (alligator) clip. Clipping of food was chosen to facilitate video recordings of the bite without the forelimbs obscuring the view of the incisors. Food clipping also prevented the rat from generating bite torque using the upper incisors and the forelimbs, which potentially would decouple jaw adductor muscle function from the biting action. A single bite series was allowed per food item to minimize FL effects in the muscle data caused by food size reduction due to repetitive biting. Figure 1B shows individual bites on different foods.

To collect gape cycle data, we recorded high speed video of all bites in a quasi-ventral view (125fps, 1024 × 1024 pixels, Fastcam SA3, Photron, San Diego, CA). Sonomicrometry data were acquired at 520 Hz (transmit pulse: 406.25 ns; inhibit delay: 1.54 mm; range-gain: 25 mm) using a digital sonomicrometer and a PC laptop running SonoLab (Sonometrics Inc., London, ON). Muscle EMG data were recorded at 100x amplification (Grass P511) with a 10 kHz high-cut and a 60 Hz notch filter engaged. Strain gauge measurements were balanced and amplified (200x; Vishay Measurements Group 2120, Raleigh, NC) and all time-varying transducer data were digitized at 4 kHz via a Powerlab 16/20 onto a PC laptop running LabChart 8.0 (ADInstruments). The animal ground was connected to the equipment ground via the sonomicrometer cable shield. A manually triggered square-wave TTL signal terminated video recordings and permitted resynchronization of time-varying transducer and gape cycle data.

### *In situ* FL ergometry

Due to *in situ* preparation and sonomicrometry transduction difficulties for two of our *in vivo* rats, we used additional rats (*N* = 4) to generate FL curves *in situ*. For these rats, sonomicrometry implantations were carried out during the ergometry preparation and EMG and force buckles were not mounted.

Directly following the *in vivo* data collection, each subject was re-induced and maintained on isoflurane, including vitals controls and monitoring as described above. The skin incision on the chin was carefully reopened, and the tendon of the superficial masseter was gently dissected free from its insertion on the rostrum and connected to the lever-arm of a servomotor (Aurora Scientific 300 C-LR) using a self-tightening knot of 1 − 0 silk and a dab pf cyanoacrylate gel (Duro) (Fig. 1C). Two pairs of stimulation electrodes were implanted and secured in place using 6 − 0 silk. Care was taken to place equivalent poles of each pair at the rostral (+) and caudal (-) ends of the muscle respectively. The insertion orientation of each electrode was normal to the fascicle plane, to avoid alterations in stimulation and recruitment as fascicles shortened against series elastic compliance. The stimulation electrode leads were flexibly suspended above the muscle preparation to minimize interference by motion during contractions. The left zygoma and ventral mandibular corpus were both firmly clamped to a customized stereotaxic frame onto which the servomotor was mounted. Muscle temperature was maintained as constant as possible using an infrared lamp and a dressing of saline soaked gauze prevented muscle desiccation during the procedure.

We acquired data on muscle stimulation intensity (V), muscle fascicle length (measured via the sonomicrometry crystals, mm), and motor force (N) along with measurements from all implanted transducers at 4 kHz via a NIDAQ (USB-6212) to a PC running IgorPro 8.0 (Wavemetrics, Lake Oswego, OR). Optimal stimulation parameters were determined for each preparation using a series of twitches with increasing voltage (6–12 volts depending on preparation; 125 pulses/s, 0.2 ms pulse width; Grass s48). Four to five 300 ms contractions from a passive tension that roughly corresponded to *L*_O_ were used remove in-series compliance and collect time-varying data from the tendon strain-gauge (V) and the motor (N) for calibration purposes (see below)^59^. Video recordings of sample tetanic contractions in two preparations were used to verify that the fixed-end compliance of the preparation was minimal (measured to be 0.7 ± 0.2 mm). Using consecutive twitches, the preparation was shortened to minimal length (*L*_REF_), which was measured from the muscle origin on the mandible to the suture knot (i.e., the entire preparation) to the nearest millimeter using calipers. Starting at *L*_REF_, we then collected the twitch and submaximal tetanic FL curves for the preparation, and the fascicles, as measured via sonomicrometry (Fig. 1D). Three minutes of rest was allowed between each tetanic stimulation to reduce effects of muscle fatigue. A final tetanic contraction at *L*_O_ was used to confirm that the preparation had lost no more than 10% of *F*_O_ during testing, which was confirmed for all preparations. Following the *in situ* experiment, the subject was euthanized (Sodium Pentobarbital, IC). The muscle was excised to record fascicle length, including the segment of the fascicles that were not spanned by the crystals, so that a segment correction could be applied to the *in vivo* data. We also measured resting muscle pennation angle and muscle mass (without the tendon) to calculate muscle physiological cross sectional area (PCSA)^61^, which was used to calculate muscle stress (N/cm^2^).

### Data extraction & analysis

#### Construction of force-length curves

We constructed force-length (FL) curves to determine twitch and submaximal tetanic optimal length (*L*_O_) from our sonomicrometry measurements of fascicle length-change and peak force (P_O_) from the motor and muscle force buckle for each SM preparation by fitting third order polynomials to the passive and active twitch and tetanic curves in IgorPRO v.8. Force (N) was expressed as stress (N/cm^2^) by dividing with muscle PCSA (Supplemental Table 1) and fascicle length (mm) was expressed as strain ((*L*-*L*_O_)/ *L*_O_). To measure the percent left-shift of tetanic *L*_O_ relative to twitch *L*_O_, we used tetanic *L*_O_ to strain-normalize twitch FL curves^25,26^. Passive FL curves were adjusted for shortening during stimulation^62^.

#### Conditioning of *in vivo* measurements

Gape cycles were digitized for usable bites in our custom confluence of DeepLabCut and XMAlab^63^. Time-varying traces of gape cycles were imported into IgorPro along with CSV data from Powerlab containing activation, length, and force measurements from the implanted transducers. Gape and instrument data were re-synchronized using the TTL square-wave signal. All subsequent signal conditioning and analyses were performed in IgorPro. Muscle EMG signals were filtered as needed using an FIR interpolation, base-line corrected and rectified. Sonomicrometry signals (V) were converted to length (mm) using a synchronized calibration recording (in SonoLab). These signals were then segment-corrected for the fascicle length not spanned by the crystal pair, and adjusted for a time-offset caused by the crystal coating epoxy^64^. The conditioned signal was smoothed using a quintic spline interpolation (s.d. = 0.01–0.05) and strain (ε = (*L*- *L*_O_)/ *L*_O_) was calculated using tetanic *L*_O_. To calibrate the tendon buckle signal (V) from *in vivo* recordings to force (N), we first regressed the time-varying gauge signal (V) against motor force (N) from the initial *in situ* tetanic contractions. Linear regressions of these two time-varying measurements for the period of tetanic force rise returned *r*^2^ values ranging from 0.87 to 0.96, which were comparable to prior validations of this technology^45,59,65^. The average regression slope (V/N) was then used to calculate time-varying *in vivo* muscle-tendon force (N), which was quintic spline interpolated as needed and baseline-corrected to zero in a period of the *in vivo* trial where the animal was at rest and no feeding occurred.

#### Extraction of in-vivo measurements

Time-varying data from the conditioned and calibrated *in vivo* measurements of SM activation, strain, and force were graphed in IgorPro. For the period of gape closing, a semi-automated procedure was used to extract the following response variable: (1) Integrated area under the rectified EMG (mV/s), (2) Fascicle starting length at the power stroke onset (*L*_MAX_), (3) Fascicle length at EMG onset (*L*_ACT_), (4) Fascicle length at peak force (*L*_MIN_), (5) Fascicle shortening strain (ε=[*L- L*_O_]/*L*_O_) during the power stroke (T_ACT_ to T_MIN_), (6) Force rise magnitude (N), and (7) Peak force (F_MAX_, N). Data were tabulated per individual (*N* = 6), cycle number (to control for effects of food size reductions across repeated bites), and food hardness. Data extraction was performed by two research assistants who were blinded to the treatment (food hardness) across trials.

#### Hypothesis testing and statistics

To test the hypothesis that activation and force production by the muscle fascicles would vary systematically with food hardness, we ran General Linear Models (GLM) in Systat v. 12. These analyses factored EMG (specifically the integrated area under the rectified electromyogram during jaw closing, in mV/s) or peak muscle force (N) as the response variables, individual as an independent effect, and food hardness (E) as the fixed factor. We interrogated the interaction term (IND*E) for statistical significance. Effect sizes of the interaction terms were calculated as the partial eta squared (η^2^) in SPSS 30.0. We also probed the strength of the EMG-force relationship across individuals and food types using Linear Least-Squares regression in Systat v. 12.

To determine if fascicle relative length (*L*/*L*_O_) at activation (*L*_ACT_) and at peak force generation (*L*_MIN_), as well as fascicle strain during the incisor biting power stroke (% *L*_O_) increased with respect to food hardness (E), we ran GLM and effect-size analyses using the design described above.

Stress-strain FL curves from the *in situ* experiments were used to determine if optimal length (*L*_O_) for rat SM varied with recruitment intensity. To determine this, we ran a GLM factoring *L*_O_ as the response variable, with stimulation protocol (twitch or submax) as a fixed factor, individual as an independent effect with interrogation of the interaction-term and computation of effect-size as above.

To determine the ranges of stresses and strains that the fascicles of the rat SM operated across during incisor biting, we plotted peak muscle stress against fascicle final strain (at peak force generation, Fig. 2B) across food items with varying hardness. Our *in vivo* fascicle operating strain data were recorded using the same sonomicrometer crystals used to measure fascicle strain *in situ*. This enabled us to superimpose *in vivo* stress-strain data onto the aggregate FL curves, expressed as stress versus strain, as measured *in situ* using submaximal stimulation.

## Supplementary Information

Below is the link to the electronic supplementary material.

Supplementary Material 1

## Data Availability

Data analyzed in this study are available from the corresponding author upon reasonable request.
