# Supplementary material for: Rat superficial masseter operates at long lengths during biting
Source: Sci Rep. 2025 Oct 30;15:37978. doi: 10.1038/s41598-025-21953-z (PMC12575779; doi:10.1038/s41598-025-21953-z)
Supplement: Supplementary file 1 — Supplementary Material 1 [file 41598_2025_21953_MOESM1_ESM.docx]

**Supplementary Materials for Konow et al 2025.**

**Supplemental table 1.** Metrics of the rats and superficial masseter muscles used in this study.

| Rat | Sex | Body mass (g) | Muscle mass (g) | Fascicle length (cm) | Fascicle pennation (°) | PCSA (cm^2^) |
| --- | --- | --- | --- | --- | --- | --- |
| 1 | M | 540 | 0.87 | 1.4 | 26 | 0.5 |
| 2 | M | 490 | 0.76 | 1.6 | 25 | 0.4 |
| 3 | F | 388 | 0.47 | 1.1 | 28 | 0.4 |
| 4 | F | 290 | 0.65 | 1.2 | 25 | 0.5 |
| 5 | M | 370 | 0.54 | 1.0 | 26 | 0.5 |
| 6 | F | 273 | 0.41 | 0.8 | 22 | 0.4 |
| 7 | M | 470 | 0.85 | 0.9 | 38 | 0.7 |
| 8 | M | 472 | 0.65 | 1.2 | 34 | 0.4 |
| 9 | M | 645 | 0.85 | 1.1 | 33 | 0.6 |
| 10 | F | 459 | 0.84 | 1.0 | 32 | 0.7 |

**Supplemental Table 2. Material properties for the food types used in this study.**

| Food | Color | Young’s modulus (MPa) | Work to fracture (J/m^2^) | Study |
| --- | --- | --- | --- | --- |
| Raisin |  | 0.22 | 307 | Reed & Ross (2010) |
| Chocolate |  | 6.86 | 118 | Afoakwa et al (2009) |
| Hazelnut |  | 12.20 | 166 | Reed & Ross (2010) |
| Almond |  | 21.57 | 246 | Reed & Ross (2010) |
| Chow |  | 50.44 | 1030 | Williams et al (2005) |

Colors correspond to figure use. Young’s modulus is material hardness (E). Work to fracture is material toughness (K).

**Supplemental Table 3. Number of bites analyzed per subject and food type.**

| Rat | Raisin | Chocolate | Hazelnut | Almond | Rat chow |
| --- | --- | --- | --- | --- | --- |
| 71 | - | 11 | - | - | 51 |
| 72 | - | 14 | - | - | - |
| 73 | - | - | - | - | 12 |
| 75 | - | 4 | 3 | 5 | 36 |
| 76 | 20 | - | - | - | 12 |
| 77 | 11 | 17 | 19 | 19 | 13 |
| Subject *N* | 2 | 4 | 2 | 2 | 5 |
| Bite *N* | 31 | 46 | 22 | 24 | 124 |
